# Intraoperative fluoroscopically assessment underestimates the posterior tibial slope in open‐wedge high tibial osteotomy

**DOI:** 10.1002/jeo2.70723

**Published:** 2026-04-14

**Authors:** Georgios Neopoulos, Alexander Berger, Patrizia Sieber, Lukas Jud, Lazaros Vlachopoulos, Sandro F. Fucentese

**Affiliations:** ^1^ Orthopaedic Department Balgrist University Hospital, University of Zurich Zurich Switzerland

**Keywords:** coronal deformity, high tibial osteotomy, posterior tibial slope, sagittal deformity, varus deformity

## Abstract

**Purpose:**

To compare intraoperative and postoperative posterior tibial slope (PTS) measurements and determine whether unintended postoperative changes occur relative to preoperative values.

**Methods:**

A total of 79 patients (81 knees) underwent open‐wedge high tibial osteotomy (owHTO) using patient‐specific instrumentation (PSI) for varus malalignment were included. PTS was assessed pre, intra and postoperatively on short lateral knee radiographs using standardised calibrated software. The hip‐knee‐ankle angle (HKA) and the medial proximal tibia angle (MPTA) were measured pre and postoperatively on weight‐bearing long leg radiographs (LLR).

**Results:**

Preoperative (10.3° [7.3−11.7°]) and intraoperative (9.1° [6.7–11.2°]) PTS measurements were significantly lower than postoperative (10.8° [8.5–13.3°]) values (*p* < 0.001). Intraoperative PTS was also significantly lower than the preoperative one (9.1° [6.7–11.2°] vs. 10.3° [7.3−11.7°]) (*p* < 0.01). Intraoperative PTS underestimated the postoperative PTS by a mean of 1.8° (95% CI: −2.1° to – 1.5°). Both HKA and MPTA changed significantly postoperatively (−7.4 ± 3.8° vs. 0.4 ± 2.6° *p* < 0.001, 84.4 ± 2.8° vs. 91.0 ± 2.8° *p* < 0.001).

**Conclusion:**

Intraoperative PTS measurements significantly underestimated postoperative PTS, by a mean of 1.8°, indicating that visual intraoperative assessment based on fluoroscopy should be interpreted with caution when used to evaluate postoperative PTS.

**Level of Evidence:**

Level IV.

**Abbreviations**
2D: two‐dimensional
3D: three‐dimensional
CI: confidence intervals
HKA: hip‐knee‐ankle angle
ICC: intraclass correlation coefficients
IQR: interquartile range
LLR: long leg radiographs
MPTA: medial proximal tibia angle
owHTO: open‐wedge high tibial osteotomy
PSI: patient‐specific instrumentation
PTS: posterior tibial slope
SD: standard deviation

## INTRODUCTION

Open‐wedge high tibial osteotomy (owHTO) is a well‐established treatment option for the correction of varus malalignment and offloading the medial compartment [5, 11, 14, 18]. While accurate coronal correction is a key factor in the clinical success of owHTO [4], maintaining the sagittal alignment by avoiding unintended changes of the posterior tibial slope (PTS) poses another challenge.

Given the challenge of maintaining sagittal alignment, a reliable PTS measurement is essential. Although several imaging modalities exist, PTS is highly sensitive to limb rotation and flexion, beam orientation, the selected tibial axis and the tibial length used for the measurement [6, 9, 15]. Using a longer tibial segment improves the measurement's accuracy and agreement, with recent evidence suggesting that PTS measured in short lateral knee radiographs may show clinically relevant error compared with measurements to the mechanical tibial axis on full‐length lateral lower leg radiographs [3, 12].

In routine practice, however, intraoperative PTS assessment relies on fluoroscopy, which is limited to short lateral knee radiographs, and it remains unclear how well intraoperative PTS reflects the postoperative one.

The aim of this study was to compare PTS measurements obtained from intraoperative fluoroscopy and postoperative short lateral knee radiographs, after owHTO using PSI. The objective was to determine whether a discrepancy exists between the two measurements. We hypothesised that the intraoperative fluoroscopic PTS assessment does not provide an accurate estimate of the postoperative PTS.

## MATERIALS AND METHODS

### Study design and patient selection

The local ethical committee approved this study (Zurich Cantonal Ethics Commission, BASEC‐Nr. 2023‐00389), and informed consent was obtained from all patients. This single‐centre study included all patients who underwent an owHTO for medial compartment degeneration or overload from October 2014 to December 2022. Inclusion criteria were: (1) intraoperative use of PSI, (2) available preoperative, intraoperative and postoperative short lateral knee radiographs with ≥10‐cm of tibial shaft visible distal to the knee joint line and (3) available weight‐bearing long leg radiographs (LLR) preoperatively and at 4.5 months postoperatively. Exclusion criteria were: (1) planned combined correction of coronal deformity and PTS and (2) concomitant ipsilateral tibial bony procedures.

### Surgical technique

All procedures were performed or supervised by two senior surgeons (L.V. and S.F.). Surgery was performed with the patient in the supine position under general or spinal anaesthesia and tourniquet control. Through an anteromedial approach, the proximal tibia was exposed, and relevant bony landmarks were prepared to allow accurate seating of the PSI. A registration/basic guide was positioned, and reference pins were inserted to define the orientation of subsequent guides. Predrilling for the plate screws was performed through integrated drill sleeves. The osteotomy guide was placed, and the open‐wedge osteotomy was performed in a PSI‐navigated fashion according to the planned osteotomy plane and predefined cutting depth. After gradual opening of the osteotomy, the predefined correction was achieved using the PSI reduction guide. Fixation was performed with a TomoFix Medial High Tibial Plate (DePuy Synthes) using the predrilled screw holes, followed by sequential insertion of screws. Fluoroscopy was used by acquiring a short lateral knee radiograph to confirm osteotomy and implant positioning, as well as visually assess the PTS relative to the tibial shaft and tibial plateau orientation. Skin was then closed over a drain.

### Radiological assessment

Radiological assessment was performed using a calibrated planning software (mediCAD®, module osteotomy; Hectec GmbH).

**Figure 1:**
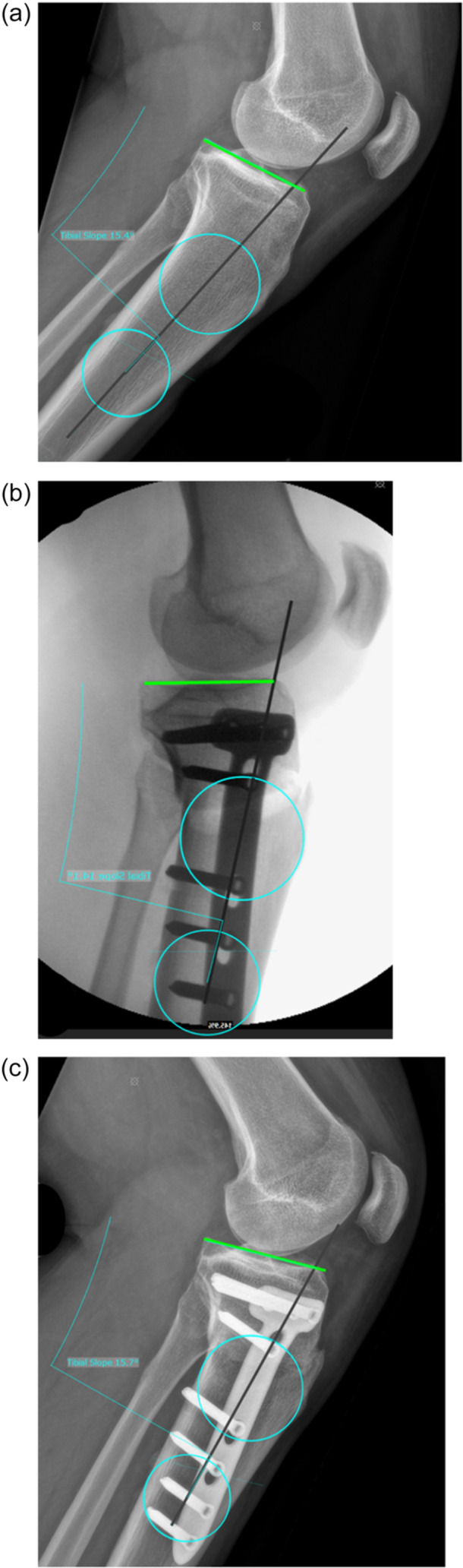
Preoperative, intraoperative and postoperative posterior tibial slope (PTS) measurements on short lateral knee radiographs. (a) preoperative short lateral knee radiograph, (b) intraoperative short lateral knee radiograph, (c) postoperative short lateral knee radiograph. To identify the proximal anatomical axis of the tibia, two circles were placed on the proximal tibial shaft with their centres at 5‐cm (proximal light blue circle) and 10‐cm (distal light blue circle) distal to the knee joint line. Each circle was drawn tangent to the anterior and posterior cortices of the proximal tibia and the line connecting their centres (black line) defined the proximal anatomical tibial axis. A line tangent to the articular surface of the medial tibial plateau (green line) was drawn. PTS was then defined as the angle between this plateau tangent and the proximal anatomical tibial axis.

Sagittal alignment: PTS was measured on preoperative, intraoperative and postoperative two‐dimensional (2D) short lateral knee radiograph. The proximal anatomical tibial axis was defined using two circles placed on the proximal tibial shaft with their centres at 5‐ and 10‐cm distal to the knee joint line. Each circle was drawn tangent to the anterior and posterior cortices of the proximal tibia, and the line connecting their centres defined the tibial axis as previously described [6, 34]. A 10‐cm distal landmark ensured consistent axis definition across the views because some intraoperative views did not include 15‐cm of tibial shaft. After identification of the proximal anatomical tibial axis, a line tangent to the articular surface of the medial tibial plateau was drawn. PTS was then defined as the angle between this plateau tangent and the proximal anatomical tibial axis (Figure 1). For interrater reliability, PTS was measured independently by two readers (A.B., P.S.) using the same software.

**Figure 2:**
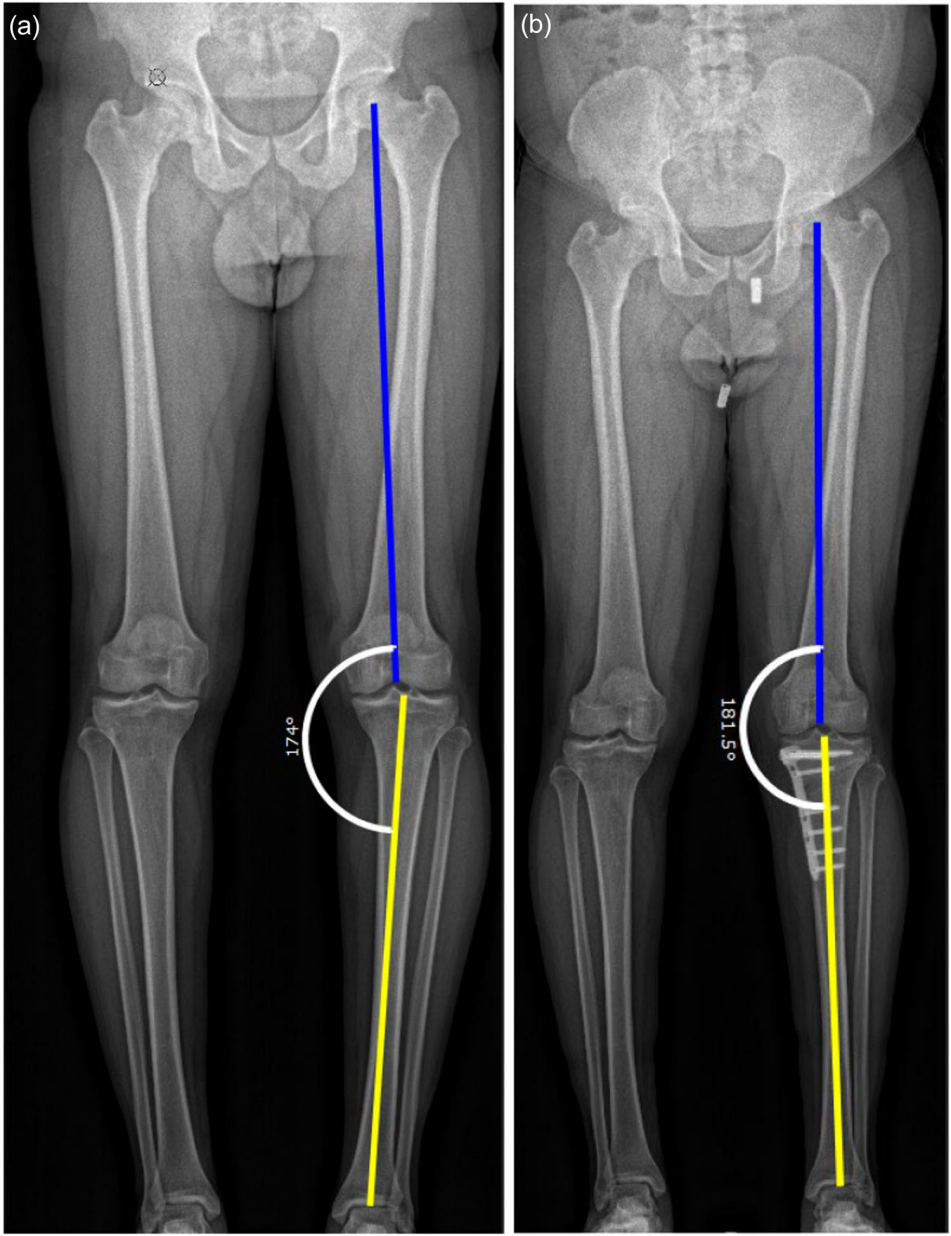
Weight‐bearing long leg radiograph with the coronal alignment of the right leg for the measurement of HKA. Weight‐bearing long leg radiograph (LLR) with the measurement of the Hip‐Knee‐Ankle angle (HKA) of the right leg preoperatively (a) and postoperatively (b). The centre of the femoral head was defined. The blue line determines the mechanical axis of the femur and the yellow line the mechanical axis of the tibia.

**Figure 3:**
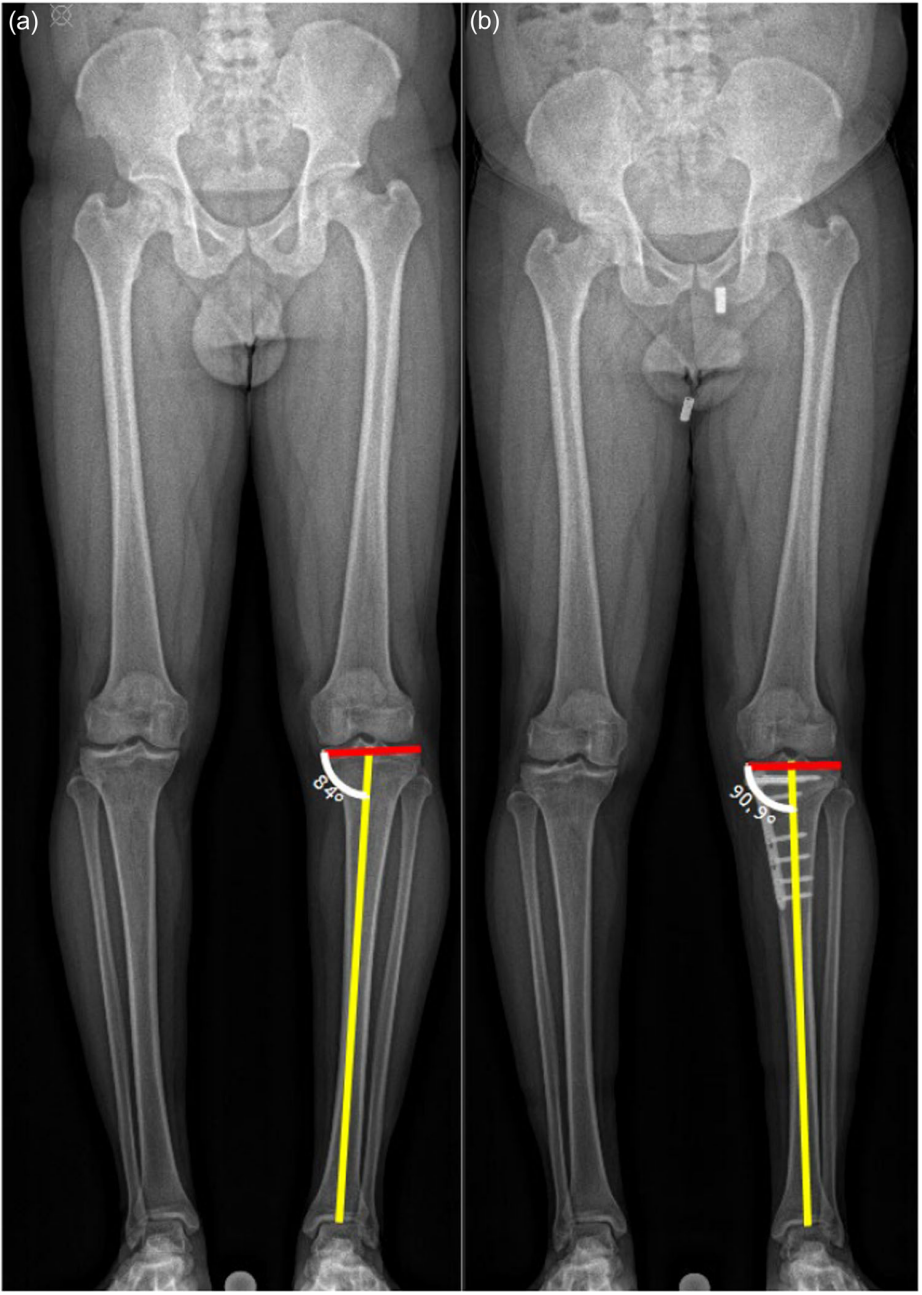
Weight‐bearing long leg radiograph with the coronal alignment of the right leg for the measurement of the MPTA. Weight‐bearing long leg radiograph (LLR) with the measurement of the mechanical proximal tibial angle (MPTA) of the right leg preoperatively (a) and postoperatively (b). The red line determines the tangent to the tibial plateau and the yellow line the mechanical axis of the tibia.

Coronal alignment: Hip‐knee‐ankle angle (HKA) was measured on weight‐bearing LLR preoperatively and at 4.5 months postoperatively (Figure 2). Positive values indicated valgus and negative values varus alignment. The medial proximal tibia angle (MPTA) was also measured on LLR at the same time points (Figure 3).

### Statistical analysis

A priori power analysis aiming for a statistical power of 0.9 and a type I error rate of 0.05 determined a required sample size of 54 patients.

Demographic data of the patients are presented using descriptive statistics. Continuous variables are reported as mean and standard deviation (SD) or median and interquartile range (IQR) as appropriate. Intraclass correlation coefficients (ICCs) with 95% confidence intervals (CIs) were calculated for the pre, intra and postoperative PTS measurements and interpreted according to Portney et al. [29]. Normality of the data distribution was assessed with the Shapiro–Wilk test. Depending on distribution, paired *t*‐test or Wilcoxon signed‐rank test were used to evaluate differences between two time points for HKA, MPTA and PTS (pre, intra and postoperative). For comparison of the PTS measurements across all three time points (pre, intra, postoperative), repeated‐measures ANOVA or the Friedman test was applied, as appropriate. A potential misinterpretation of intraoperative versus postoperative PTS was further assessed using the Hodges–Lehmann median difference and Bland‐Altman plots. All statistical analyses were performed in RStudio for Mac (Posit software). Significance was set at *p* < 0.05.

## RESULTS

**Figure 4:**
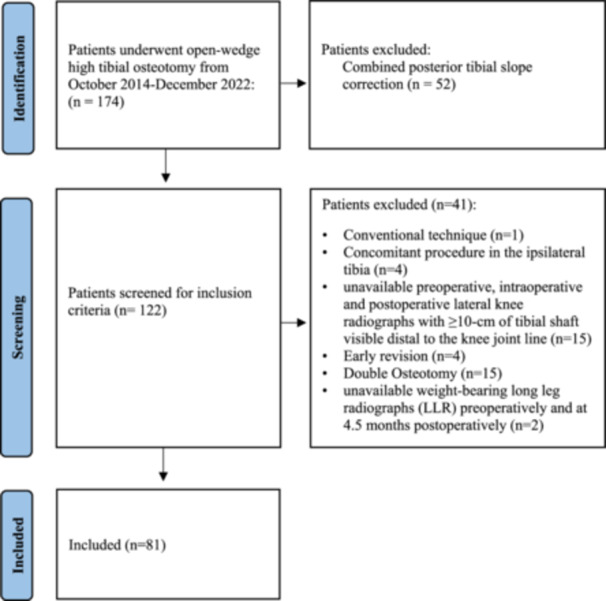
STROBE‐flow diagram. STROBE, strengthening the reporting of observational studies in epidemiology.

Eighty‐one knees (43 left knees, 38 right knees) from 79 patients (14 female, 65 male) were included in the study (Figure 4). Detailed demographics are presented in Table 1.

**Table 1:** Demographic characteristics.

Demographic characteristics	
*n*	81 knees, 79 patients
Age, average (range), years	44.5 (21–63)
Female gender, *n* (%)	14 (18)
Left side, *n* (%)	43 (53)
BMI, average ± SD, kg/m^2^	29.6 ± 5

Abbreviations: BMI, body mass index; SD, standard deviation.

Coronal alignment: HKA and MPTA changed significantly from preoperative to postoperative situation in all 81 knees (both *p* < 0.05). Values are summarised in Table 2.

**Table 2:** Pre and postoperative HKA and MPTA.The values are given in average value, SD and range.

	Preopeative	Postoperative	*p*‐value
HKA, average ± SD (range), °	−7.4 ± 3.8 (−24.2, −1.7)	0.4 ± 2.7 (−5.8, 6.0),	<0.001
MPTA, average ± SD (range), °	84.4 ± 2.8 (69.2, 89.7)	91.0 ± 2.8 (82.2, 99.4)	<0.001

Abbreviations: HKA, hip‐knee‐ankle angle; MPTA, medial proximal tibia angle; SD, standard deviation.

**Figure 5:**
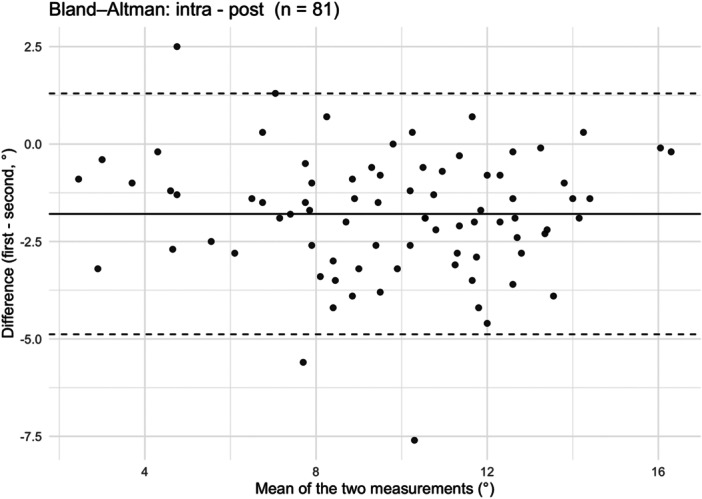
Bland‐Altman plot comparing intraoperative and postoperative posterior tibial slope (PTS) measurements. Each point represents one knee (*n* = 81). The *x*‐axis shows the mean of intraoperative and postoperative PTS measurements, and the *y*‐axis the difference between them. The solid horizontal line indicates the mean difference of intraoperative and postoperative PTS measurements across all cases, which is −1,8°, meaning the intraoperative PTS systematically underestimates postoperative PTS. The dashed horizontal lines represent the limits of agreement (mean difference ± 1.96 standard deviation).

PTS measurements: Significant differences were observed between each two‐time‐points: preoperative vs intraoperative (*p* < 0.01), intraoperative versus postoperative (*p* < 0.001) and preoperative versus postoperative (*p* < 0.001). The overall comparison between all three time points was also significantly different (*p* < 0.001). The Hodges–Lehmann and the Bland–Altman analysis showed that intraoperative PTS measurements underestimated the postoperative PTS by 1.8° (95% CI: −2.1° to – 1.5°). Detailed results are presented in Table 3 and the Bland‐Altman plot is shown in Figure 5. The absolute deviation between intraoperative and postoperative PTS was <1° in 25 knees (30.8%), ≥1° but < 3° in 40 knees (49,4%), ≥3° but < 5° in 14 knees (17.3%) and ≥5° in 2 knees (2.5%).

**Table 3:** Pre, intra and postoperative PTS.

	PTS	*p*‐value
Preop. versus intraop., median (IQR), °	10.3 (7.3−11.7) versus 9.1 (6.7–11.2)	<0.01^a^
Preop. versus postop., median (IQR), °	10.3 (7.3−11.7) versus 10.8 (8.5–13.3)	<0.001^a^
Intraop. versus postop., median (IQR), °	9.1 (6.7–11.2) versus 10.8 (8.5–13.3)	<0.001^a^
preop. versus intraop. versus postop., median (IQR), °	10.3 (7.3−11.7) versus 9.1 (6.7–11.2) versus 10.8 (8.5–13.3)	<0.001^b^

Abbreviations: intraop., intraoperative; IQR: interquartile range, postop., postoperative; Preop., preoperative; PTS, posterior tibial slope.

^a^ Wilcoxon‐signed‐rank test.

^b^ Friedman test.

The ICC demonstrated good reliability for preoperative (0.891 [95% CI: 0.831–0.929]) and intraoperative (0.878 [95% CI: 0.817–0.919]) PTS measurements, and excellent reliability for postoperative (0.904 [95% CI: 0.839–0.941]) PTS measurements.

## DISCUSSION

The key finding of this study is that intraoperative PTS assessment based on short lateral fluoroscopic knee radiographs did not reflect the postoperative PTS. Intraoperative PTS systematically underestimated the postoperative PTS by 1.8°. From a practical standpoint, this questions the accuracy of intraoperative visual PTS assessment using fluoroscopy during owHTO. Accordingly, the hypothesis of the study could be confirmed.

During owHTO, besides accurate correction of the coronal alignment, preserving the native PTS represents a major challenge with several studies reporting unintended PTS changes, which can disrupt anteroposterior knee instability and increase stress on the anterior cruciate ligament (ACL) [2, 8, 13, 17, 20, 23, 32, 33]. Technical intraoperative factors, such as incomplete posterior corticotomy and anterior plate positioning, have been implicated to influence this PTS change [28, 30]. While our cohort showed a significant increase in postoperative PTS compared with preoperative values, the magnitude was smaller than commonly reported in the literature (10.3° [7.3°−11.7°] vs. 10.8° [8.5°−13.3°]) [25, 27]. This might be attributed to the preoperative three‐dimensional (3D) planning and the use of PSI, which has been associated with improved accuracy and reduced unintended PTS changes compared with conventional techniques [11, 25, 35].

Accurate measurement of the PTS itself is another challenge. Prior works have shown that PTS measurements vary depending on the imaging modality and the length of the tibia used to define its anatomical axis, with longer tibial length improving measurement accuracy [9, 12, 15, 22]. Recent data demonstrated that PTS measured on routine short lateral knee radiographs can show substantial error when compared with PTS measured on full‐length lateral lower‐leg radiographs, supporting their use when precise PTS quantification is required [3]. However, the 10‐cm landmark/method has been validated as a practical and reliable alternative with <1° deviation compared to the 15‐cm method used on short lateral knee radiographs [16].

During owHTO, intraoperatively PTS control is based on fluoroscopic short lateral knee radiographs, but the accuracy of the intraoperative PTS measurement in predicting the postoperative PTS has not been clarified. Our study addresses this gap, demonstrating that despite using the same measurement method pre, intra and postoperatively, the intraoperative PTS consistently underestimated the postoperative one by 1.8°. Particularly, deviations of more than 3° occurred in 19.8% of knees, and between 1° and 3° in 49.4%, despite the use of PSI applied in all cases, indicating that PSI does not fully eliminate unintended PTS changes in owHTO. This suggests that intraoperative fluoroscopy, likely influenced by factors such as limb positioning and projection geometry (condylar overlap, beam centering and distance) [21], may be prone to systematic bias and is not a valid assessment tool to predict postoperative PTS.

The clinical relevance of a mean underestimation of 1.8° remains uncertain, as no clinical meaningful cut‐offs for intraoperative‐postoperative PTS discrepancy have been defined for owHTO. Nevertheless, given that higher PTS has been associated with ACL graft failure as well as revision risk and threshold have been determined in this context [7, 10, 19, 26, 31], this underestimation proven in our study may be worth considering in patients with high preoperative PTS or instability.

The present study should be interpreted in light of its potential limitations. First, the PTS was measured on short lateral knee radiographs, despite the fact, that using full‐length lateral lower‐leg radiographs is recommended as it improves the accuracy of PTS measurement [3, 12] and despite the variability in PTS measurements among radiographs, CT and MRI [1, 24]. Additionally, we did not validate PTS against CT or 3D imaging; thus, measurement accuracy could not be determined. However, the same imaging technique and measurement protocol were deliberately applied pre, intra and postoperatively, meaning that potential systematic errors are likely to be uniform across all time points. Therefore, our conclusions apply to fluoroscopic short lateral knee radiographs and its ability to predict postoperative PTS also on short knee lateral radiographs. Second, the limb position and subsequently the superimposition of the femoral condyles and the overlapping of the tibia plateaus was not controlled intraoperatively and was less consistent than on pre and postoperative radiographs, which may have contributed to measurement discrepancies. A prospective approach with predefined fluoroscopic criteria, including condylar superimposition and standardised beam centering and distance, as well as an additional baseline intraoperative lateral radiograph before osteotomy could potentially clarify the contribution of projection‐related error. Third, this study did not investigate clinical correlates of PTS changes or measurement discrepancies (e.g., postoperative extension deficit or patient‐reported outcomes), as this was outside the scope of the predefined radiographic research question. Although no preoperative extension deficit was present in our cohort, the radiographic follow‐up of 4.5 months would not be a safe time point to draw conclusions on extension deficiency. Lastly, this was a retrospective single‐centre cohort of owHTO using PSI, which may limit generalisability to other settings and to conventional techniques.

## CONCLUSION

Intraoperative PTS measurements significantly underestimated postoperative PTS by a mean of 1.8°, indicating that visual intraoperative assessment based on fluoroscopy should be interpreted with caution when used to evaluate postoperative PTS.

## AUTHOR CONTRIBUTIONS

**Georgios Neopoulos**: Conceptualisation; methodology; writing—original draft preparation; writing—review and editing; statistical analysis; project administration. **Alexander Berger**: Methodology; data curation; writing—review and editing; statistical analysis. **Patrizia Sieber**: Data curation; writing—review and editing. **Lukas Jud**: Writing—review and editing. **Lazaros Vlachopoulos**: Writing—review and editing; supervision. **Sandro F. Fucentese**: Conceptualisation; writing—review and editing; supervision. All authors have read and agreed to the published version of the manuscript.

## CONFLICT OF INTEREST STATEMENT

The authors declare no conflicts of interest.

## ETHICS STATEMENT

Ethical review and approval of the local ethical committee were obtained for this study (Zurich Cantonal Ethics Commission, BASEC‐Nr. 2023‐00389). Each patient included in this study signed the written informed consent.

## Supporting information


**Supplementary Material:** STROBE Checklist.

## Data Availability

The data that support the findings of this study are available from the corresponding author upon reasonable request.
