# Supplementary material for: Intraoperative fluoroscopically assessment underestimates the posterior tibial slope in open‐wedge high tibial osteotomy
Source: J Exp Orthop. 2026 Apr 14;13(2):e70723. doi: 10.1002/jeo2.70723 (PMC13078137; doi:10.1002/jeo2.70723)
Supplement: Supplementary file 1 — Supplementary Material: STROBE Checklist. [file JEO2-13-e70723-s001.doc]

STROBE Statement—Checklist of items that should be included in reports of ***cohort studies***

|  | Item No | Recommendation |
| --- | --- | --- |
| **Title and abstract** | 1 | (*a*) Indicate the study’s design with a commonly used term in the title or the abstract. Lines 1-2 |
|  |  | (*b*) Provide in the abstract an informative and balanced summary of what was done and what was found. Lines 7-25 |
| Introduction | | |
| Background/rationale | 2 | Explain the scientific background and rationale for the investigation being reported. Lines 27-42 |
| Objectives | 3 | State specific objectives, including any prespecified hypotheses. Lines 43-47. |
| Methods | | |
| Study design | 4 | Present key elements of study design early in the paper. Lines 49-59 |
| Setting | 5 | Describe the setting, locations, and relevant dates, including periods of recruitment, exposure, follow-up, and data collection. Lines 49-59 |
| Participants | 6 | (*a*) Give the eligibility criteria, and the sources and methods of selection of participants. Describe methods of follow-up. Lines 49-59 |
|  |  | (*b*) For matched studies, give matching criteria and number of exposed and unexposed |
| Variables | 7 | Clearly define all outcomes, exposures, predictors, potential confounders, and effect modifiers. Give diagnostic criteria, if applicable. Lines 77-95 |
| Data sources/ measurement | 8* | For each variable of interest, give sources of data and details of methods of assessment (measurement). Describe comparability of assessment methods if there is more than one group. Lines 77-95. |
| Bias | 9 | Describe any efforts to address potential sources of bias. Lines 77-95 |
| Study size | 10 | Explain how the study size was arrived at. Lines 97-98 |
| Quantitative variables | 11 | Explain how quantitative variables were handled in the analyses. If applicable, describe which groupings were chosen and why. Lines 99-112 |
| Statistical methods | 12 | (*a*) Describe all statistical methods, including those used to control for confounding Lines 99-112 |
|  |  | (*b*) Describe any methods used to examine subgroups and interactions. Not applicable |
|  |  | (*c*) Explain how missing data were addressed. Not applicable |
|  |  | (*d*) If applicable, explain how loss to follow-up was addressed. Not applicable |
|  |  | (*e*) Describe any sensitivity analyses. Not applicable |
| Results | | |
| Participants | 13* | (a) Report numbers of individuals at each stage of study—eg numbers potentially eligible, examined for eligibility, confirmed eligible, included in the study, completing follow-up, and analysed. Lines 114-118. |
|  |  | (b) Give reasons for non-participation at each stage. Not applicable |
|  |  | (c) Consider use of a flow diagram. See Figure 4 |
| Descriptive data | 14* | (a) Give characteristics of study participants (eg demographic, clinical, social) and information on exposures and potential confounders. Lines 114-118. Table 1 |
|  |  | (b) Indicate number of participants with missing data for each variable of interest. Not applicable |
|  |  | (c) Summarise follow-up time (eg, average and total amount). Lines 114-118. |
| Outcome data | 15* | Report numbers of outcome events or summary measures over time. Not applicable |
| Main results | 16 | (*a*) Give unadjusted estimates and, if applicable, confounder-adjusted estimates and their precision (eg, 95% confidence interval). Make clear which confounders were adjusted for and why they were included. Lines 116-128. |
|  |  | (*b*) Report category boundaries when continuous variables were categorized. Lines 126-128. |
|  |  | (*c*) If relevant, consider translating estimates of relative risk into absolute risk for a meaningful time period. Not applicable |
| Other analyses | 17 | Report other analyses done—eg analyses of subgroups and interactions, and sensitivity analyses. Not applicable |
| Discussion | | |
| Key results | 18 | Summarise key results with reference to study objectives. Lines 134-139. |
| Limitations | 19 | Discuss limitations of the study, taking into account sources of potential bias or imprecision. Discuss both direction and magnitude of any potential bias. Lines 179-203. |
| Interpretation | 20 | Give a cautious overall interpretation of results considering objectives, limitations, multiplicity of analyses, results from similar studies, and other relevant evidence. Lines 140-178 |
| Generalisability | 21 | Discuss the generalisability (external validity) of the study results. Lines 140-178 |
| Other information | | |
| Funding | 22 | Give the source of funding and the role of the funders for the present study and, if applicable, for the original study on which the present article is based. Line 208. |

*Give information separately for exposed and unexposed groups.

**Note:** An Explanation and Elaboration article discusses each checklist item and gives methodological background and published examples of transparent reporting. The STROBE checklist is best used in conjunction with this article (freely available on the Web sites of PLoS Medicine at http://www.plosmedicine.org/, Annals of Internal Medicine at http://www.annals.org/, and Epidemiology at http://www.epidem.com/). Information on the STROBE Initiative is available at http://www.strobe-statement.org.
